# Psychological factors demonstrate the largest incremental predictive value in a multi-domain machine learning model for secondary injury risk after ACL reconstruction

**DOI:** 10.3389/fpsyg.2026.1832229

**Published:** 2026-05-15

**Authors:** Shengjie Xiong, Yongtie Wu, Shunmei Liu

**Affiliations:** Department of Sports Rehabilitation, Zunyi First People's Hospital (Third Affiliated Hospital of Zunyi Medical University), Zunyi, Guizhou, China

**Keywords:** anterior cruciate ligament reconstruction, Kinesiophobia, machine learning, secondary injury prediction, SHAP interpretability

## Abstract

**Background:**

Secondary injury after anterior cruciate ligament (ACL) reconstruction, defined as ipsilateral graft rerupture or contralateral ACL rupture, remains a clinical challenge. Current prediction models predominantly fail to capture this multifactorial risk. In this study, we developed a multi-domain machine learning model to predict the risk of secondary injury.

**Methods:**

This retrospective cohort study included 487 patients who underwent primary ACL reconstruction. Thirty predictor variables spanning demographic, magnetic resonance imaging (MRI), gait analysis, isokinetic strength, and psychological domains were collected at a standardized 6-month postoperative follow-up. Five machine learning algorithms were evaluated using a nested cross-validation scheme, with SHAP analysis and domain ablation applied for interpretability.

**Results:**

Sixty-four patients (13.1%) sustained secondary injuries (ipsilateral graft re-rupture or contralateral ACL rupture). Logistic regression achieved the best discriminative performance (AUC = 0.739, 95% CI: 0.672–0.806) and calibration (Brier score = 0.106), although no statistically significant difference was observed between logistic regression and random forest (corrected paired *t*-test, *p* = 0.72). SHAP analysis of both the random forest and logistic regression models identified the TSK score and PHQ-9 as the most influential individual predictors; bootstrap resampling indicated moderate stability of these rankings. Domain ablation confirmed that the psychological domain provided the largest incremental predictive contribution, with its removal producing the greatest performance decrement (AUC decline: 0.031, 95% CI: 0.007–0.058), whereas the removal of the MRI or gait domains did not reduce model performance. All models demonstrated high negative predictive values (0.89–0.94) but limited precision (0.21–0.26), indicating that most patients flagged as high risk would not sustain a secondary injury. Decision curve analysis indicated a net clinical benefit in the 0.05–0.15 threshold range, supporting a low-threshold screening rather than a diagnostic application.

**Conclusion:**

A multi-domain machine learning model identified patients at elevated secondary injury risk with acceptable discrimination and calibration. Kinesiophobia and depressive symptoms showed the largest incremental predictive contributions among the domains examined, suggesting that systematic psychological screening within postoperative rehabilitation warrants further investigation in prospective and externally validated studies.

## 1 Introduction

Anterior cruciate ligament (ACL) tears are among the most common musculoskeletal injuries encountered in orthopedic sports medicine, with an annual incidence of approximately 68.6 per 100,000 individuals and substantially higher rates among competitive athletes (Sanders et al., 2016; Montalvo et al., 2019). Surgical reconstruction is the standard of care for restoring knee stability and facilitating the return to pre-injury activity levels. In the United States alone, more than 200,000 ACL reconstruction (ACLR) procedures are performed annually (Mall et al., 2014). Despite advances in surgical techniques, graft selection, and rehabilitation protocols over the past two decades, secondary injury to the reconstructed knee or contralateral limb remains a clinically significant challenge. Graft re-rupture rates of 6–15% and contralateral ACL injury in an additional 5–12% of patients within the first 5 years after surgery have been reported (Wiggins et al., 2016; Paterno et al., 2014). Secondary injuries impose substantial physical, psychological, and financial burdens on patients, frequently resulting in prolonged rehabilitation, diminished long-term knee function, and accelerated progression of posttraumatic osteoarthritis (Luc et al., 2014).

Numerous investigations have sought to identify the risk factors for secondary ACL injury, and the existing literature implicates a broad spectrum of demographic, anatomical, biomechanical, and behavioral determinants. Younger age, female sex, return to high-level pivoting sports, and early return to competition have been associated with an elevated re-injury risk in prospective cohort studies (Wiggins et al., 2016; Webster and Feller, 2016). Biomechanical investigations employing three-dimensional motion capture have demonstrated that patients undergoing ACLR frequently exhibit aberrant movement patterns during dynamic tasks. Such patterns, including excessive knee valgus moment, reduced knee flexion angle at initial contact, and persistent gait asymmetry, may predispose the reconstructed or contralateral knee to re-injury (Paterno et al., 2010; Di Stasi et al., 2013). Isokinetic strength testing has similarly shown that persistent quadriceps and hamstring weakness, commonly quantified using the limb symmetry index (LSI), is associated with altered landing mechanics and an increased injury risk (Grindem et al., 2016; Kyritsis et al., 2016). Quantitative MRI techniques, such as T2 mapping, have enabled the non-invasive assessment of early cartilage degeneration and graft maturation, providing objective biomarkers that may reflect the biological readiness of the reconstructed knee (Li et al., 2011; Biercevicz et al., 2015). Despite a substantial body of domain-specific evidence, existing prediction models have predominantly relied on variables from a single clinical domain and failed to capture the multifactorial nature of secondary injury risk.

Despite their frequent omission from quantitative risk models, a growing body of evidence has highlighted the importance of psychological factors in post-ACLR outcomes. Kinesiophobia, defined as excessive and debilitating fear of physical movement arising from perceived re-injury vulnerability, was measured using the Tampa Scale for Kinesiophobia (TSK; range 17–68, with higher scores indicating greater kinesiophobia). It has been identified as a robust predictor of delayed return to sports and self-reported functional limitations (Ardern et al., 2013; Kvist et al., 2005). Depressive symptom burden, quantified using instruments such as the Patient Health Questionnaire-9 (PHQ-9; range 0–27, with higher scores indicating greater depressive symptom severity), has been associated with reduced rates of return to pre-injury activity, diminished adherence to rehabilitation programs, and impaired self-efficacy (Lentz et al., 2015; Brewer et al., 2006). The ACL-Return to Sport after Injury (ACL-RSI) scale (range 0–100, with higher scores indicating greater psychological readiness to resume sports) has been shown to predict actual return-to-sport outcomes in several prospective studies (Webster et al., 2008; Ardern et al., 2014). However, these constructs have rarely been integrated into quantitative prediction frameworks alongside objective biomechanical and imaging data. Integrating psychological measures with structural, functional, and biomechanical assessments is conceptually compelling but methodologically challenging, as the resulting high-dimensional feature space and complex nonlinear interactions among variables exceed the capacity of traditional regression-based approaches.

Machine learning offers a principled framework for modeling high-dimensional, heterogeneous clinical data, with growing applications in orthopedic outcome prediction (Kunze et al., 2021; Martin et al., 2022). Ensemble methods, such as random forest, gradient boosting (XGBoost, LightGBM), and support vector machines, can identify nonlinear relationships and higher-order interactions among predictors without requiring explicit specification of functional forms, in contrast to traditional logistic regression (Nordhausen, 2009). Model-agnostic interpretability methods, such as SHapley Additive exPlanations (SHAP) enable the transparent quantification of individual feature contributions, addressing the longstanding criticism that machine learning models lack interpretability (Lundberg and Lee, 2017). Nevertheless, the application of machine learning to secondary ACL injury prediction remains limited, and no prior study has simultaneously incorporated MRI-derived biomarkers, instrumented gait analysis, isokinetic strength testing, and validated psychological questionnaires within a unified predictive framework. Accordingly, this study aimed to (1) develop and internally validate a multi-domain machine learning prediction model for secondary injury risk following ACLR, (2) evaluate the discriminative performance and calibration of five candidate algorithms, and (3) identify the most influential predictor domains and individual features through SHAP-based interpretability analysis and domain ablation experiments.

## 2 Methods

### 2.1 Study design and participants

This retrospective cohort study included 487 patients who underwent primary unilateral anterior cruciate ligament reconstruction (ACLR) at a single tertiary sports medicine center. Patients who underwent ACLR between January 2018 and December 2022 were screened for eligibility. The inclusion criteria were as follows: (1) age 16–60 years at the time of surgery; (2) primary ACLR with autograft (bone-patellar tendon-bone, hamstring tendon, or quadriceps tendon); (3) a minimum of 12 months of postoperative follow-up with complete clinical assessment data; and (4) availability of postoperative MRI, gait analysis, isokinetic strength testing, and psychological questionnaire data collected at the standardized follow-up visit. The exclusion criteria were revision ACLR, multi-ligament reconstruction, bilateral ACL injury, and incomplete data records. The institutional review board approved the study protocol, and written informed consent was obtained from all the participants. Because complete data across all five assessment domains were required for inclusion (criterion 4), no missing values were present in the analytic dataset; accordingly, no imputation or missing data handling was performed.

### 2.2 Outcome definition

The primary outcome was secondary injury, defined as either ipsilateral graft rerupture or contralateral ACL rupture, confirmed by clinical examination (positive Lachman and pivot shift tests) and MRI during the follow-up period. This composite endpoint is hereafter referred to as “secondary injury.” Of the 487 patients enrolled, 64 (13.1%) had secondary injury events.

### 2.3 Prediction horizon and temporal framework

All predictor variables were collected at a standardized postoperative assessment visit conducted 6 months (±2 weeks) after ACLR. This timepoint was selected because it corresponds to the phase during which return-to-sport clearance decisions are typically initiated, and all five assessment domains (MRI, gait, isokinetic strength, and psychological questionnaires) are clinically indicated at this stage. The median total follow-up duration was 28 months (interquartile range [IQR], 20–38 months). Among the 64 patients who sustained a secondary injury, the median time from the 6-month assessment to the injury event was 14 months (IQR: 8–22). The majority of secondary injuries (53 of 64, 82.8%) occurred after the patient returned to sports. Therefore, the prediction model estimates secondary injury risk beyond the 6-month postoperative assessment, a time point at which most predictor values are available and clinically actionable. It should be noted that some psychological, strength, and gait variables measured at 6 months may change between the assessment and the eventual injury event; accordingly, the model is best characterized as postoperative risk profiling at a single time point rather than dynamic prospective prediction.

### 2.4 Predictor variables

Thirty predictor variables across five clinical domains were collected during a standardized 6-month postoperative assessment visit.

Demographic and surgical variables included age, sex, body mass index (BMI), smoking status, graft type (bone-patellar tendon-bone, hamstring tendon, or quadriceps tendon), concomitant meniscus injury, concomitant cartilage injury, duration from injury to surgery (weeks), and pre-injury Tegner activity level.

MRI parameters were obtained from quantitative postoperative imaging and included T2 mapping values (reflecting cartilage composition and early degeneration), tunnel widening (mm), graft signal intensity (normalized to the posterior cruciate ligament), bone edema volume (cm^3^), cartilage thickness (mm), and meniscus score (0–3 ordinal grading).

Gait analysis metrics were derived from three-dimensional motion capture during level-ground walking and included the peak knee flexion angle during the stance phase, peak external knee valgus moment (normalized to body weight × height), gait asymmetry index (inter-limb difference), step length difference (cm), and vertical loading rate (body weight per second).

Muscle strength indices were obtained from isokinetic dynamometry at 60°/s and included the quadriceps LSI, hamstring LSI, hamstring-to-quadriceps (H/Q) ratio, quadriceps peak torque (Nm), and hamstring peak torque (Nm).

Three-dimensional gait analysis was performed using a Vicon T40S motion capture system (Vicon Motion Systems Ltd., Oxford, UK) with an eight-camera configuration and a modified Plug-in Gait full-body reflective marker set. Kinematic and kinetic data were sampled at 100 Hz and 1,000 Hz, respectively, and synchronized using Vicon Nexus 2.12 software. Ground reaction forces were recorded by two AMTI OR6-7 force plates (Advanced Mechanical Technology Inc., Watertown, MA, USA) embedded in a 10-meter walkway. Participants walked barefoot at a self-selected speed. A minimum of five valid trials (defined as clean single-foot force plate strikes) were collected per participant, and ensemble averages were used for analysis. Isokinetic strength testing was performed using a Biodex System 4 Pro dynamometer (Biodex Medical Systems Inc., Shirley, NY, USA). Participants performed five maximal concentric reciprocal knee flexion–extension repetitions at an angular velocity of 60°/s, following three submaximal familiarization repetitions. Gravity correction was applied using the limb weight measured at full knee extension. Peak torque (Nm) was recorded as the highest value across all maximal repetitions for each muscle group.

Psychological assessments included the ACL-RSI scale (0–100, with higher scores indicating greater psychological readiness), TSK score (17–68, with higher scores indicating greater kinesiophobia, i.e., worse psychological burden), Knee Injury and Osteoarthritis Outcome Score quality-of-life subscale (KOOS-QoL, 0–100, with higher scores indicating better quality of life), 36-Item Short Form Health Survey mental component summary (SF-36 Mental, 0–100, with higher scores indicating better mental health), and PHQ-9 score (0–27, with higher scores indicating greater depressive symptom severity, i.e., worse psychological burden). In summary, higher TSK and PHQ-9 scores reflected greater psychological distress, whereas higher ACL-RSI, KOOS-QoL, and SF-36 Mental scores reflected better psychological status.

The variable “return to sport” was not included in the predictor set, despite its availability in the dataset. This decision was made because return to sport is a necessary precondition for sustaining a pivoting-related secondary injury, and its inclusion as a predictor would introduce temporal confounding and potential data leakage in a study design in which the exposure (return to sport) and outcome (secondary injury during sport) cannot be temporally disentangled (Grindem et al., 2018). The univariate association between return to sport and secondary injury (82.8% vs. 66.0%, *p* = 0.011) likely reflects this inherent temporal dependency rather than a genuine causal predictive relationship.

### 2.5 Statistical analysis of baseline characteristics

Continuous variables are presented as mean ± standard deviation and were compared between groups using independent-sample *t*-tests. Categorical variables are presented as frequencies and percentages and were compared using chi-square tests (or Fisher’s exact test when expected cell counts were <5). Because the univariate comparisons encompassed 30 variables and were exploratory in nature, the Benjamini–Hochberg false discovery rate (FDR) procedure was applied at a significance level of 0.05 to account for the multiple comparisons.

### 2.6 Machine learning model development

Five candidate algorithms were evaluated: logistic regression (LR), random forest (RF), support vector machine (SVM) with a radial basis function kernel, extreme gradient boosting (XGBoost), and light gradient boosting machine (LightGBM). All continuous features were standardized to zero mean and unit variance. Standardization parameters (mean and standard deviation) were computed exclusively within each outer training fold and applied to the corresponding held-out test fold to prevent information leakage from the test set into the preprocessing step.

A nested cross-validation scheme was employed to obtain unbiased performance estimates and concurrently optimize the hyperparameters (Varma and Simon, 2006). The outer loop used a stratified five-fold cross-validation for performance evaluation. Within each outer training fold, an inner three-fold stratified cross-validation with grid search and AUC as the optimization criterion was used for the hyperparameter tuning. The three-fold inner scheme was chosen to balance the computational cost with the constraint of approximately 51 positive events per outer training fold, ensuring a minimum of approximately 17 events per inner fold. The hyperparameter search spaces were as follows: LR (regularization parameter C: 0.01, 0.1, 1, 10); RF (number of trees: 100, 200; maximum depth: 3, 5, 7; minimum samples per leaf: 10, 20); SVM (C: 0.1, 1, 10; kernel: radial basis function; gamma: scale); XGBoost (number of trees: 100, 200; maximum depth: 3, 5; learning rate: 0.01, 0.1; minimum child weight: 5, 10; L2 regularization lambda: 1, 5); LightGBM (same grid as XGBoost). Conservative tree depth limits (maximum 5–7) and enlarged minimum leaf sizes (10–20) were deliberately chosen to mitigate overfitting given the limited number of positive events (*n* = 64) relative to the number of predictors (approximately 2.1 events per variable), a constraint addressed further in the Discussion. The final selected hyperparameter for logistic regression across the five outer folds was *C* = 1.0. No explicit class imbalance correction (e.g., SMOTE or class weighting) was applied; the low event rate was addressed through conservative regularization, restricted tree complexity, and post-hoc Platt scaling calibration.

Platt scaling (sigmoid calibration) using three-fold within-fold cross-validation was applied to all models that did not natively yield well-calibrated probabilities (RF, SVM, XGBoost, and LightGBM) (Platt, 2000). Logistic regression directly optimizes the log-likelihood and often yields reasonably calibrated probabilities; therefore, it was not subjected to post-hoc calibration in the present analysis.

Optimal classification thresholds were identified by maximizing Youden’s *J* statistic (sensitivity + specificity − 1) on each training fold and applied to the corresponding held-out test fold (Youden, 1950).

### 2.7 Model evaluation metrics

Discriminative performance was assessed using the area under the receiver operating characteristic curve (AUC) and the area under the precision-recall curve (AUPRC), the latter being particularly informative for imbalanced datasets (Saito and Rehmsmeier, 2015). Bootstrap 95% confidence intervals (2,000 iterations) were computed for both the AUC and AUPRC from pooled out-of-fold predictions. Probability calibration was evaluated using the Brier score, with the naïve baseline Brier score (0.114 for this dataset) serving as the reference. Calibration curves were constructed by plotting the observed event rates against the predicted probabilities in decile bins. Additional classification metrics, including sensitivity, specificity, precision, negative predictive value (NPV), and F1 score, were derived from pooled cross-validation predictions at Youden’s J-optimized threshold. Confusion matrices were assembled from out-of-fold predictions across all five folds, a procedure explicitly distinguished from the training set performance (Steyerberg and Harrell, 2016). To assess whether the performance differences between the models were statistically meaningful, a corrected resampled paired *t*-test (Varma and Simon, 2006) was applied to the fold-level AUC estimates of the two best-performing models.

### 2.8 Model interpretability

SHAP analysis was applied to both the random forest and logistic regression models trained on the complete dataset to quantify individual feature contributions (Lundberg and Lee, 2017). Because the SHAP values were computed on models trained on the full dataset rather than within the cross-validation framework, these results should be interpreted as exploratory characterizations of feature importance rather than validated findings. To assess the stability of the SHAP-derived feature rankings, a bootstrap resampling procedure (500 iterations) was performed. In each iteration, a bootstrap sample of the full dataset was drawn with replacement, the model was refitted, SHAP values were recomputed, and the frequency with which each feature appeared in the top-3 and top-5 rankings was recorded.

In addition, a multivariable logistic regression model was fitted to the complete standardized dataset, yielding odds ratios (ORs) with 95% bootstrap confidence intervals (CIs; 2,000 iterations) for each predictor. Prior to the multivariate analysis, pairwise Spearman correlations were computed across all 30 predictors. Variables exhibiting pairwise correlations exceeding |*r*| = 0.70 with another predictor within the same clinical domain were excluded from the logistic regression, retaining the more clinically interpretable variable from each pair. The specific collinear pairs, the retained variable from each pair, and the rationale for retention are detailed in Supplementary Table S3. This procedure resulted in 15 variables being entered into the multivariable logistic regression model, and all 30 features were retained in the machine learning models.

### 2.9 Domain ablation analysis

A leave-one-domain-out ablation analysis was performed to quantify the relative incremental contributions of each clinical domain. The logistic regression model was retrained using five-fold cross-validation after successively removing each of the five predictor domains (MRI, gait, strength, psychological, and demographic), and the resulting AUC was compared with that of the full model. To quantify the uncertainty around the ablation decrements, a bootstrap procedure (500 iterations) was applied: in each iteration, a bootstrap sample was drawn, the full and ablated models were both retrained via five-fold cross-validation, and the AUC difference was recorded. The 2.5th and 97.5th percentiles of the resulting distribution provided 95% confidence intervals for each domain’s contribution.

### 2.10 Decision curve analysis

Decision curve analysis (DCA) was performed to assess the clinical utility of the best-performing model across a range of threshold probabilities (Vickers and Elkin, 2006). The net benefit was computed and compared with the default strategies of treating all patients and none. A risk distribution plot was constructed to identify the optimal risk stratification threshold.

### 2.11 Software and reproducibility

All analyses were performed using Python 3.11.9 with scikit-learn 1.4.2, XGBoost 2.0.3, LightGBM 4.3.0, and SHAP 0.44.1 software. A random seed of 42 was fixed for all the stochastic procedures to ensure reproducibility.

## 3 Results

### 3.1 Baseline characteristics

The analysis included 487 patients, of whom 64 (13.1%) experienced secondary injury (42 ipsilateral graft re-ruptures and 22 contralateral ACL ruptures) during follow-up. The demographic and clinical characteristics stratified by injury status are presented in Table 1. The mean age of the cohort was 27.90 ± 6.47 years, and 305 participants (62.6%) were male. No significant between-group differences were observed in age (27.08 ± 6.26 vs. 28.02 ± 6.50 years, *p* = 0.284), sex (*p* = 0.908), BMI (23.81 ± 3.18 vs. 24.21 ± 3.21 kg/m^2^, *p* = 0.350), smoking status (*p* = 0.576), graft type (*p* = 0.717), concomitant meniscus injury (*p* = 1.000), or concomitant cartilage injury (*p* = 0.541).

**Table 1:** Baseline demographic and clinical characteristics stratified by secondary injury status.

Variable	No injury (*n* = 423)	Secondary injury (*n* = 64)	*p* value
Age (years)	28.02 ± 6.50	27.08 ± 6.26	0.284
BMI (kg/m^2^)	24.21 ± 3.21	23.81 ± 3.18	0.350
Time to surgery (wk)	9.44 ± 7.20	9.83 ± 8.98	0.699
MRI T2 mapping (ms)	34.65 ± 8.55	38.88 ± 8.39	<0.001
Tunnel widening (mm)	1.76 ± 0.84	2.16 ± 0.79	<0.001
Graft signal intensity	2.10 ± 0.72	2.26 ± 0.60	0.083
Bone edema volume (cm^3^)	5.38 ± 3.63	6.41 ± 4.09	0.037
Cartilage thickness (mm)	2.41 ± 0.50	2.29 ± 0.53	0.082
Peak knee flexion (deg)	58.27 ± 8.10	60.09 ± 6.50	0.088
Peak valgus moment (Nm/BW × Ht)	0.34 ± 0.12	0.39 ± 0.12	0.012
Gait asymmetry index (%)	8.04 ± 4.95	10.16 ± 5.50	0.002
Step length difference (cm)	2.12 ± 1.58	2.79 ± 1.71	0.002
Vertical loading rate (BW/s)	45.51 ± 12.37	43.87 ± 13.49	0.329
Quadriceps LSI (%)	82.63 ± 12.47	77.53 ± 12.54	0.002
Hamstring LSI (%)	86.14 ± 10.48	80.50 ± 10.38	<0.001
H/Q ratio	0.59 ± 0.12	0.53 ± 0.13	<0.001
Quadriceps peak torque (Nm)	152.81 ± 39.34	147.10 ± 44.59	0.288
Hamstring peak torque (Nm)	86.40 ± 22.57	82.75 ± 23.86	0.232
ACL-RSI (higher = better readiness)	59.65 ± 17.84	51.96 ± 17.22	0.001
TSK score (higher = greater kinesiophobia)	37.83 ± 7.88	42.85 ± 7.00	<0.001
KOOS-QoL (higher = better QoL)	63.35 ± 15.91	55.15 ± 17.70	<0.001
SF-36 mental (higher = better mental health)	69.54 ± 14.10	67.24 ± 14.20	0.226
PHQ-9 (higher = worse depressive symptoms)	3.54 ± 1.92	5.09 ± 2.46	<0.001
Tegner pre-injury	6.30 ± 1.62	6.34 ± 1.64	0.859
Sex, male—*n* (%)	264 (62.4%)	41 (64.1%)	0.908
Smoking—*n* (%)	64 (15.1%)	12 (18.8%)	0.576
Graft type—*n* (%)			0.717
BPTB	215 (50.8%)	31 (48.4%)	
Hamstring tendon	163 (38.5%)	24 (37.5%)	
Quadriceps tendon	45 (10.6%)	9 (14.1%)	
Meniscus injury—*n* (%)	145 (34.3%)	22 (34.4%)	1.000
Cartilage injury—*n* (%)	63 (14.9%)	12 (18.8%)	0.541
Return to sport—*n* (%)^†^	279 (66.0%)	53 (82.8%)	0.011

BPTB, bone-patellar tendon-bone; LSI, limb symmetry index; H/Q, hamstring-to-quadriceps; ACL-RSI, ACL-Return to Sport after Injury scale; TSK, Tampa Scale for Kinesiophobia; KOOS-QoL, Knee Injury and Osteoarthritis Outcome Score quality-of-life subscale; SF-36 Mental, 36-Item Short Form Health Survey mental component summary; PHQ-9, Patient Health Questionnaire-9.

^†^Return to sport was not included in predictive modeling due to temporal confounding (see Methods Section 2.4).

Several MRI parameters differed significantly between groups. Patients with secondary injury had higher T2 mapping values (38.88 ± 8.39 vs. 34.65 ± 8.55 ms, *p* < 0.001) and greater tunnel widening (2.16 ± 0.79 vs. 1.76 ± 0.84 mm, *p* < 0.001); bone edema volume was also significantly greater in the injury group (6.41 ± 4.09 vs. 5.38 ± 3.63 cm^3^, *p* = 0.037). Gait analysis revealed significantly higher peak knee valgus moment (0.39 ± 0.12 vs. 0.34 ± 0.12 Nm/BW × Ht, *p* = 0.012), gait asymmetry index (10.16 ± 5.50 vs. 8.04 ± 4.95%, *p* = 0.002), and step length difference (2.79 ± 1.71 vs. 2.12 ± 1.58 cm, *p* = 0.002) in the secondary injury group. Muscle strength indices were consistently lower in the injury group: quadriceps LSI (77.53 ± 12.54 vs. 82.63 ± 12.47%, *p* = 0.002), hamstring LSI (80.50 ± 10.38 vs. 86.14 ± 10.48%, *p* < 0.001), and H/Q ratio (0.53 ± 0.13 vs. 0.59 ± 0.12, *p* < 0.001). Psychological assessments revealed significantly higher TSK scores (indicating greater kinesiophobia; 42.85 ± 7.00 vs. 37.83 ± 7.88, *p* < 0.001), higher PHQ-9 scores (indicating greater depressive symptoms; 5.09 ± 2.46 vs. 3.54 ± 1.92, *p* < 0.001), lower ACL-RSI scores (indicating poorer psychological readiness; 51.96 ± 17.22 vs. 59.65 ± 17.84, *p* = 0.001), and lower KOOS-QoL scores (indicating worse quality of life; 55.15 ± 17.70 vs. 63.35 ± 15.91, *p* < 0.001) in the secondary injury group. SF-36 Mental scores did not differ significantly between groups (67.24 ± 14.20 vs. 69.54 ± 14.10, *p* = 0.226). Return to sport was more prevalent in the secondary injury group (82.8% vs. 66.0%, *p* = 0.011); however, this variable was excluded from predictive modeling due to temporal confounding concerns as described in Section 2.4.

### 3.2 Model discrimination performance

**Figure 1:**
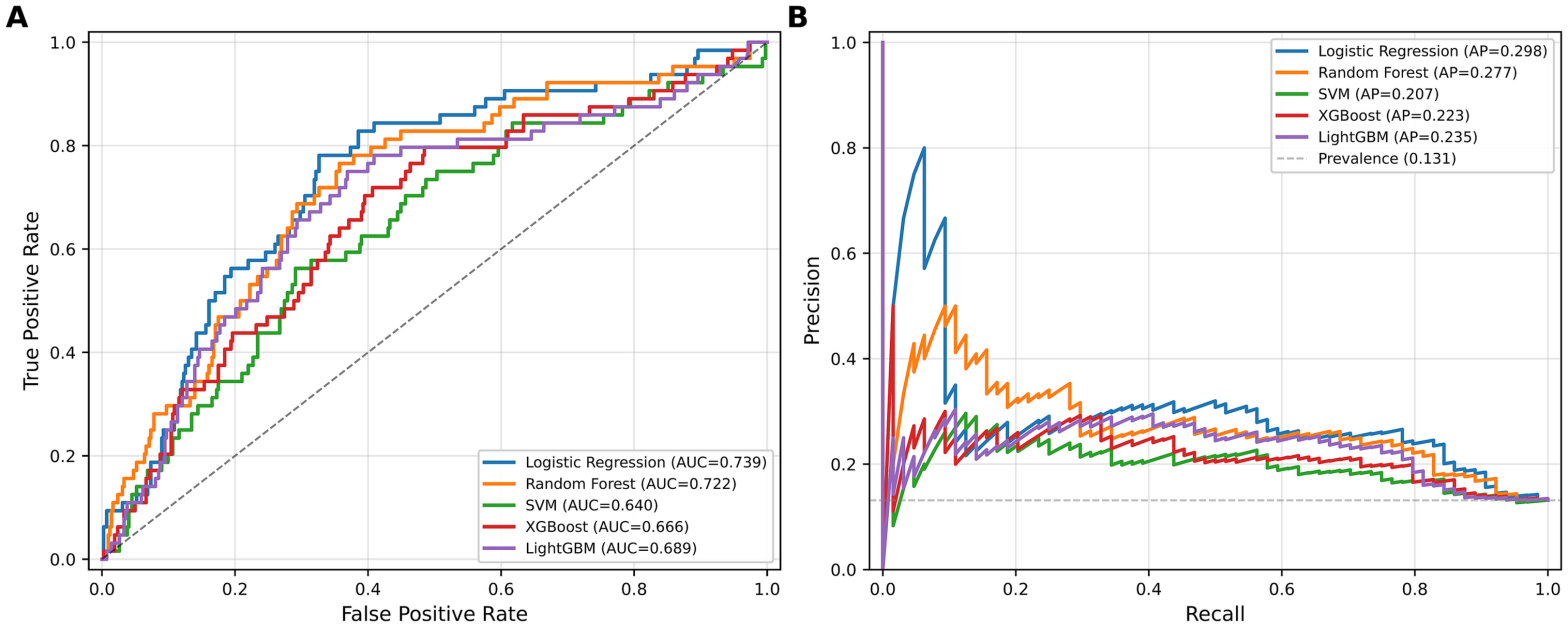
Receiver operating characteristic (ROC) curves **(A)** and precision-recall curves **(B)** for five machine learning models evaluated using pooled five-fold cross-validation predictions. The dashed diagonal line in panel A represents random classification. The dashed horizontal line in panel B indicates the prevalence of secondary injury (13.1%).

The pooled cross-validation performance metrics for all five models are presented in Table 2. Logistic regression achieved the highest AUC of 0.739 (95% CI: 0.672–0.806; five-fold mean: 0.742 ± 0.081), followed by random forest (AUC = 0.722, 95% CI: 0.657–0.787; five-fold mean: 0.738 ± 0.072), LightGBM (AUC = 0.689, 95% CI: 0.618–0.760; five-fold mean: 0.722 ± 0.090), XGBoost (AUC = 0.666, 95% CI: 0.593–0.739; five-fold mean: 0.691 ± 0.083), and SVM (AUC = 0.640, 95% CI: 0.567–0.713; five-fold mean: 0.641 ± 0.071). A corrected resampled paired *t*-test comparing the fold-level AUCs of logistic regression and random forest yielded *t* = 0.38, *p* = 0.72, indicating no statistically significant performance difference between the two models. The fold-level AUC values for all models are reported in Supplementary Table S1. In the precision-recall space, logistic regression also achieved the highest AUPRC (0.298), followed by random forest (0.277), LightGBM (0.235), XGBoost (0.223), and SVM (0.207), all of which substantially exceeded the naïve baseline AUPRC of 0.131. At Youden’s J-optimized thresholds, logistic regression achieved a sensitivity of 0.719 and specificity of 0.683, and random forest achieved a sensitivity of 0.609 and specificity of 0.723. The precision values ranged from 0.214 (XGBoost) to 0.256 (logistic regression), indicating that approximately three in four patients flagged as high-risk would not sustain a secondary injury. All models demonstrated high negative predictive values (0.893–0.941). The ROC and precision-recall curves are shown in Figure 1.

**Table 2:** Pooled five-fold cross-validation performance metrics for five machine learning models.

Model	AUC (95% CI)	AUPRC	Brier score	Sensitivity	Specificity	Precision	NPV	F1
Logistic regression	0.739 (0.672–0.806)	0.298	0.1060	0.719	0.683	0.256	0.941	0.377
Random forest	0.722 (0.657–0.787)	0.277	0.1064	0.609	0.723	0.250	0.924	0.355
LightGBM	0.689 (0.618–0.760)	0.235	0.1109	0.500	0.752	0.234	0.909	0.318
XGBoost	0.666 (0.593–0.739)	0.223	0.1119	0.531	0.704	0.214	0.909	0.305
SVM	0.640 (0.567–0.713)	0.207	0.1125	0.359	0.806	0.219	0.893	0.272

AUC, area under the receiver operating characteristic curve; AUPRC, area under the precision-recall curve; NPV, negative predictive value; SVM, support vector machine. 95% CIs for AUC were computed from 2,000 bootstrap iterations of pooled out-of-fold predictions. Naïve baseline Brier score = 0.114; naïve baseline AUPRC = 0.131. Sensitivity, specificity, precision, NPV, and F1 score were derived from pooled out-of-fold predictions at the Youden’s J-optimized threshold. A corrected resampled paired *t*-test comparing LR and RF fold-level AUCs yielded *p* = 0.72.

### 3.3 SHAP feature importance analysis

SHAP analysis was performed on both the random forest and logistic regression models trained on the full dataset; these results are exploratory and should be interpreted with a low events-per-variable ratio in mind.

**Figure 2:**
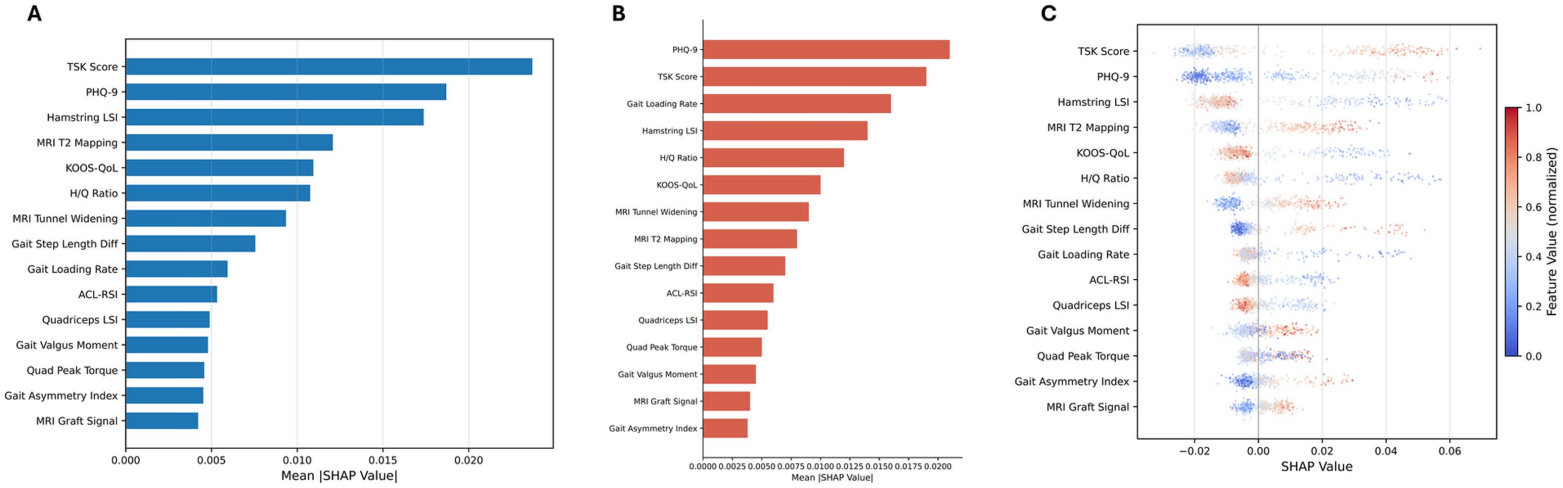
SHAP analysis of the random forest and logistic regression models. **(A)** Bar plot of mean absolute SHAP values for the top 15 features from the random forest model, ranked by importance. **(B)** Bar plot of mean absolute SHAP values for the top 15 features from the logistic regression model, ranked by importance. **(C)** Beeswarm plot showing the distribution and direction of SHAP values for each feature from the random forest model, with color indicating normalized feature values (blue = low, red = high). Note: SHAP values were computed on models trained on the full dataset and should be interpreted as exploratory.

For the random forest model, SHAP analysis identified the TSK score as the most influential predictor (mean |SHAP| = 0.024), followed by the PHQ-9 (0.019), hamstring LSI (0.017), MRI T2 mapping (0.012), and KOOS-QoL (0.011) (Figure 2A). For the logistic regression model, SHAP analysis identified the PHQ-9 as the most influential predictor (mean |SHAP| = 0.021), followed by the TSK score (0.019), vertical loading rate (0.016), hamstring LSI (0.014), and H/Q ratio (0.012) (Figure 2B). The two models converged in identifying the TSK score and PHQ-9 as the top two features, although their relative ranking differed, consistent with the capacity of the random forest to capture nonlinear effects.

The SHAP beeswarm plot (Figure 2C) confirmed the directionality of these associations: higher TSK and PHQ-9 scores and lower hamstring LSI were each associated with an elevated predicted secondary injury risk. Higher MRI T2 mapping values and lower KOOS-QoL scores similarly increased the risk predictions.

Bootstrap stability analysis (500 resamples) indicated that for the random forest model, TSK appeared among the top-3 features in 87.4% of resamples, PHQ-9 in 83.2%, and hamstring LSI in 71.6%. For the logistic regression model, the PHQ-9 appeared in the top three in 90.2% of resamples, TSK in 85.8%, and loading rate in 68.4% (Supplementary Table S2). These results suggest moderate stability of the top-ranked features, although the lower-ranked features showed considerable variability across resamples, consistent with the limited statistical power of our dataset.

### 3.4 Model calibration

**Figure 3:**
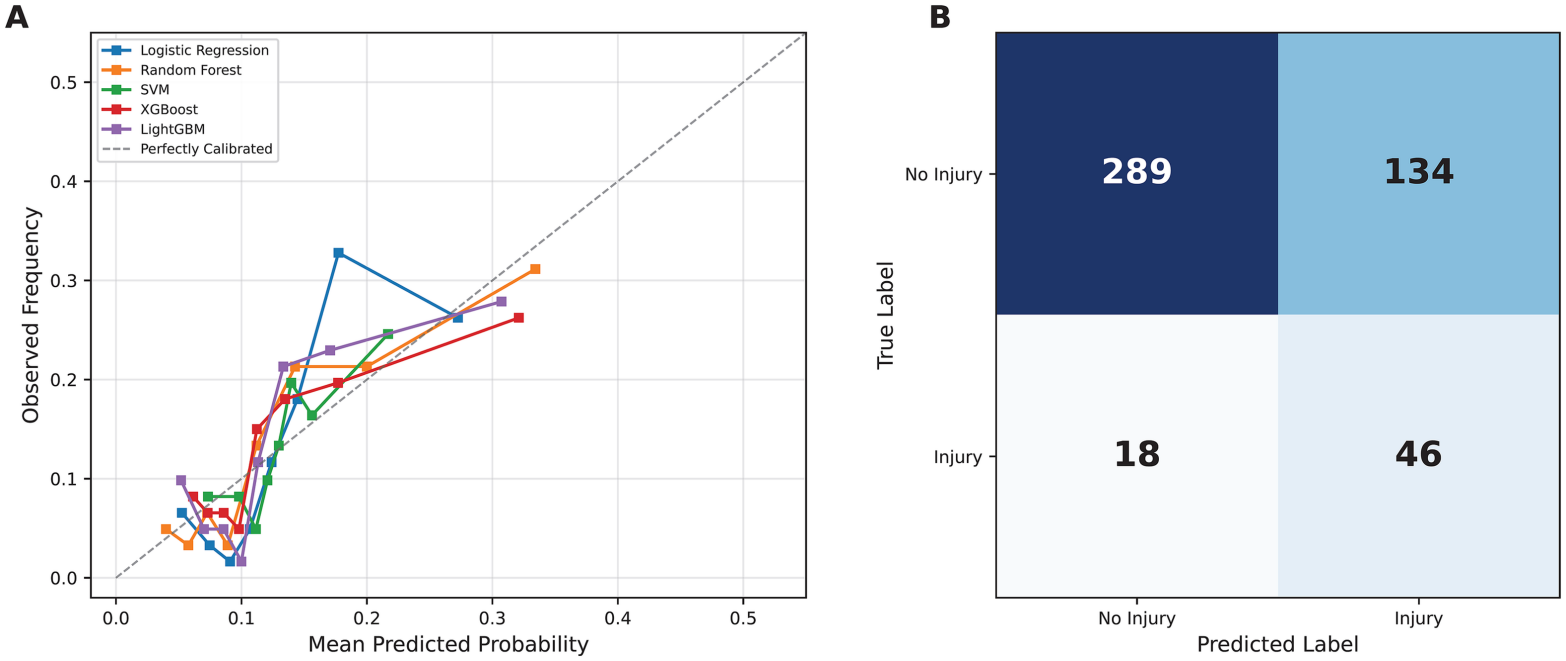
Calibration curves **(A)** and pooled five-fold cross-validation confusion matrix for the best-performing logistic regression model **(B)**. Panel A displays the relationship between predicted probabilities and observed event frequencies for each model. Panel B presents the confusion matrix at the Youden’s J-optimized threshold.

Following Platt scaling calibration, all five models achieved Brier scores below the naïve baseline (0.114). Logistic regression and random forest demonstrated the lowest Brier scores (0.1060 and 0.1064, respectively), followed by LightGBM (0.1109), XGBoost (0.1119), and SVM (0.1125). The calibration curves (Figure 3A) indicated that logistic regression and random forest most closely approximated the ideal diagonal, particularly in the clinically relevant low-to-moderate probability range (0–0.30). SVM showed a greater deviation from perfect calibration at higher predicted probabilities. The pooled five-fold cross-validation confusion matrix for logistic regression (Figure 3B) yielded 46 true positives, 289 true negatives, 134 false positives, and 18 false negatives across 487 total out-of-fold predictions, consistent with the reported sensitivity (0.719) and specificity (0.683) values.

### 3.5 Domain ablation analysis

**Figure 4:**
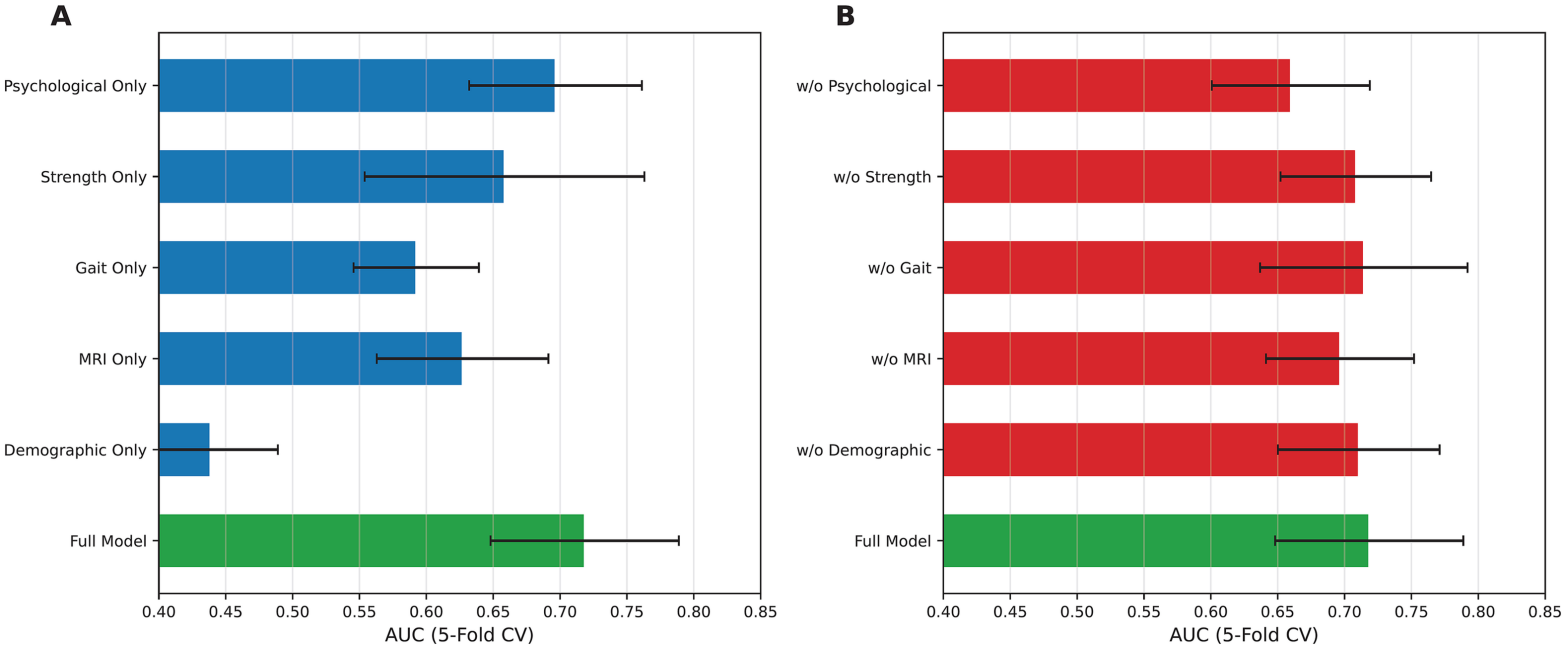
Domain ablation analysis. **(A)** AUC values for logistic regression models trained using each individual clinical domain alone compared with the full multi-domain model. **(B)** Leave-one-domain-out analysis showing model performance after removing each clinical domain from the full logistic regression model. All values are AUCs from five-fold cross-validation; the full model AUC was 0.742. Error bars represent 95% bootstrap confidence intervals from 500 iterations. Lower AUC after domain removal indicates greater incremental predictive contribution of the omitted domain.

Domain ablation analysis (Figure 4) quantified the incremental contribution of each clinical domain to the model performance. Removing the psychological domain produced the largest performance decrement (AUC decrease from 0.742 to 0.711, a decline of 0.031; 95% CI for decrement: 0.007–0.058), followed by the removal of the strength domain (AUC decrease to 0.732, decline of 0.010; 95% CI: −0.012 to 0.034). The removal of the MRI or gait domains did not reduce the AUC (both increased to 0.752; 95% CIs for the change included zero), indicating that these domains did not show additional incremental predictive value in this dataset and modeling framework beyond what was already captured by the remaining features. Similarly, the removal of the demographic domain did not impair performance (AUC increased to 0.766), consistent with the absence of significant univariate associations for most demographic variables. The bootstrap confidence interval for the psychological domain ablation effect excluded zero, supporting the interpretation that this domain provided a statistically meaningful incremental contribution; however, the confidence intervals for all other domains included zero, indicating that their individual contributions could not be distinguished from chance in this study sample.

### 3.6 Psychological variable distributions

**Figure 5:**
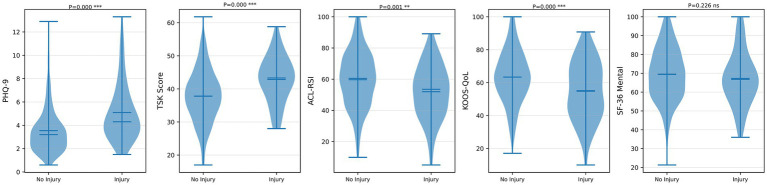
Violin plots comparing the distributions of five psychological variables between the secondary injury and no-injury groups. *P* values were derived from independent-samples *t*-tests. For scale direction: higher TSK and PHQ-9 scores indicate greater psychological distress; higher ACL-RSI, KOOS-QoL, and SF-36 mental scores indicate better psychological status. PHQ-9, Patient Health Questionnaire-9; TSK, Tampa Scale for Kinesiophobia; ACL-RSI, ACL-Return to Sport after Injury scale; KOOS-QoL, Knee Injury and Osteoarthritis Outcome Score quality-of-life subscale; SF-36 mental, 36-Item Short Form Health Survey mental component summary.

The distributions of the five psychological variables, stratified by secondary injury status, are shown in Figure 5. The PHQ-9 (higher = worse), TSK (higher = worse), ACL-RSI (higher = better), and KOOS-QoL (higher = better) scores showed statistically significant between-group differences (all *p* < 0.01), whereas the SF-36 Mental (higher = better) score did not (*p* = 0.226). The SF-36 Mental subscale was nevertheless retained in the predictive model, as univariate non-significance does not preclude incremental predictive value in a multivariable context, and exclusion of variables based on univariate testing alone can introduce selection bias in multivariable prediction models.

### 3.7 Risk stratification and decision curve analysis

**Figure 6:**
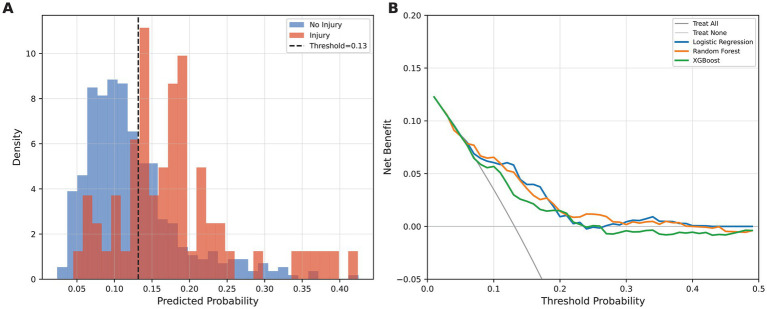
Risk stratification and decision curve analysis. **(A)** Distribution of predicted risk scores for the secondary injury and no-injury groups, with the optimal threshold (0.135) indicated by the vertical dashed line. **(B)** Decision curve analysis comparing the net benefit of the logistic regression model against the default strategies of treating all patients and treating no patients.

The risk score distributions for both groups are shown in Figure 6A, with a separation between the secondary injury and no-injury groups. The optimal classification threshold identified by Youden’s J statistic was 0.135. At this threshold, 71.9% of secondary injury events were correctly identified, whereas the specificity was maintained at 68.3%. Decision curve analysis (Figure 6B) demonstrated the net benefit of the logistic regression model over both the “treat all” and “treat none” default strategies across threshold probabilities of approximately 0.05–0.15. Beyond a threshold of approximately 0.20, the net benefit approached or fell below zero, indicating that the model’s clinical utility is primarily in low-threshold screening applications rather than high-threshold confirmatory diagnosis.

### 3.8 Multi-metric model comparison

A radar chart comparing all five models across the six performance dimensions is presented in Supplementary Figure S1. These dimensions included AUC, AUPRC, sensitivity, specificity, F1 score, and calibration (1 − Brier score). Logistic regression achieved the highest values for AUC, AUPRC, sensitivity, and F1, whereas SVM showed the lowest sensitivity among the five models.

### 3.9 Multivariable logistic regression analysis

**Figure 7:**
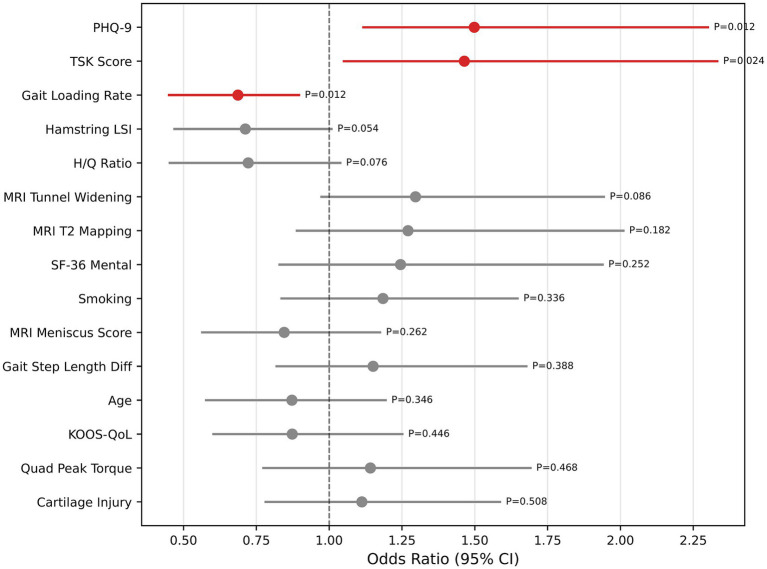
Forest plot of odds ratios with 95% bootstrap confidence intervals from multivariable logistic regression. Red markers denote statistically significant predictors (*p* < 0.05); gray markers denote non-significant predictors.

The multivariable logistic regression results are presented in Figure 7 and Table 3. PHQ-9 exhibited the highest odds ratio (OR = 1.50, 95% CI: 1.12–2.30, *p* = 0.013), followed by TSK score (OR = 1.46, 95% CI: 1.05–2.33, *p* = 0.026). The loading rate was identified as a protective factor (OR = 0.69, 95% CI: 0.46–0.92, *p* = 0.018). Hamstring LSI (OR = 0.71, 95% CI: 0.46–1.00, *p* = 0.053) and H/Q ratio (OR = 0.72, 95% CI: 0.46–1.04, *p* = 0.081) showed trends toward a protective effect that did not reach statistical significance. MRI T2 mapping (OR = 1.27, 95% CI: 0.87–1.99, *p* = 0.194) and tunnel widening (OR = 1.30, 95% CI: 0.97–1.96, *p* = 0.081) showed positive associations with secondary injury risk, which were also non-significant.

**Table 3:** Multivariable logistic regression results: odds ratios with 95% bootstrap confidence intervals (2,000 iterations).

Variable	OR	95% CI	*p* value
PHQ-9	1.498	1.123–2.298	0.013
TSK score	1.464	1.052–2.325	0.026
Loading rate	0.687	0.457–0.915	0.018
Hamstring LSI	0.712	0.463–1.004	0.053
H/Q ratio	0.722	0.463–1.038	0.081
Tunnel widening	1.296	0.974–1.959	0.081
MRI T2 mapping	1.271	0.869–1.992	0.194
SF-36 mental	1.245	0.839–1.911	0.234
Smoking	1.185	0.811–1.631	0.351
Meniscus score	0.846	0.557–1.168	0.260
Step length diff	1.151	0.820–1.666	0.369
Age	0.872	0.580–1.200	0.356
KOOS-QoL	0.873	0.603–1.250	0.418
Quadriceps peak torque	1.142	0.779–1.727	0.475
Cartilage injury	1.112	0.772–1.575	0.486

OR, odds ratio; CI, confidence interval; PHQ-9, Patient Health Questionnaire-9; TSK, Tampa Scale for Kinesiophobia; LSI, limb symmetry index; H/Q, hamstring-to-quadriceps; KOOS-QoL, Knee Injury and Osteoarthritis Outcome Score quality-of-life subscale; SF-36 Mental, 36-Item Short Form Health Survey mental component summary.

## 4 Discussion

This study developed and internally validated a multi-domain machine learning prediction model for secondary injury risk following ACLR, integrating MRI-derived biomarkers, three-dimensional gait analysis, isokinetic muscle strength assessment, and psychological questionnaires, within a unified predictive framework. Logistic regression achieved the best discriminative performance (AUC = 0.739) and probability calibration (Brier score = 0.106) among the five candidate algorithms, although the difference relative to random forest was not statistically significant (*p* = 0.72). SHAP analysis and domain ablation experiments consistently identified the psychological domain as providing the largest incremental predictive contribution. The clinical and methodological implications of these findings warrant detailed consideration in future studies.

The central finding is the relatively large incremental contribution of psychological variables to secondary ACL injury prediction compared with other clinical domains. TSK score and PHQ-9 ranked as the two most important features by SHAP analysis in both the random forest and logistic regression models, and multivariable logistic regression confirmed both as independent risk factors (OR = 1.46, *p* = 0.026 and OR = 1.50, *p* = 0.013, respectively). Domain ablation corroborated this finding: removal of the psychological domain produced the largest performance decrement (AUC decline of 0.031, 95% CI: 0.007–0.058), while removal of the MRI or gait domains left performance unchanged or marginally improved it. These findings are consistent with an expanding body of literature underscoring the psychological dimensions of ACL recovery. Ardern et al. (2013) identified kinesiophobia measured by the TSK as an important modifiable predictor of failure to return to pre-injury sport, independent of knee function and time from surgery. Lentz et al. (2015) demonstrated that elevated depressive symptoms at 3 months post-ACLR predicted worse patient-reported outcomes at 2 years, independent of surgical and demographic factors. The mechanistic pathway through which psychological distress may contribute to secondary injury plausibly involves multiple interacting processes, though these pathways were not directly tested in the present study. Kinesiophobia may induce compensatory movement strategies characterized by excessive muscular co-contraction and reduced knee flexion during dynamic tasks, which could increase mechanical loading on the graft and articular cartilage (Hartigan et al., 2013). Concurrently, depressive symptoms may impair adherence to rehabilitation, engagement in neuromuscular training, and acquisition of protective proprioceptive control (Brewer et al., 2006; Everhart et al., 2015). While speculative in the context of the current study, these considerations support the potential value of incorporating validated psychological screening instruments such as the TSK and PHQ-9 into standard postoperative assessment protocols, with targeted interventions, such as cognitive behavioral therapy, graded exposure programs, and mental performance consultation, offered to patients identified as at risk (Coronado et al., 2018). However, the clinical benefit of such interventions for secondary injury prevention remains to be established in prospective trials.

It is important to note that the present findings indicate the largest incremental predictive value of the psychological domain within the specific context of this dataset and the modeling framework. This should not be interpreted as evidence that psychological factors are causally dominant or mechanistically primary in the pathway to secondary ACL injury. The relatively modest ablation decrement (0.031 AUC units) and moderate bootstrap stability of individual feature rankings indicate that these findings require replication in larger, prospective cohorts before strong clinical recommendations can be made.

The second important finding is the divergence between the SHAP-based feature importance from the random forest model and the odds ratios from the multivariable logistic regression. MRI T2 mapping ranked fourth in RF SHAP importance but yielded a non-significant odds ratio (OR = 1.27, *p* = 0.194), and hamstring LSI ranked third in RF SHAP analysis but produced a borderline odds ratio (OR = 0.71, *p* = 0.053). Notably, the LR-based SHAP analysis produced a ranking more consistent with the logistic regression odds ratios, with the PHQ-9 ranking first and the loading rate ranking third. This divergence does not reflect analytic inconsistency; rather, it reflects the fundamentally different quantities estimated by linear and nonlinear models. SHAP values derived from the random forest capture nonlinear and interaction effects that are unavailable to logistic regression. For example, MRI T2 mapping values may exhibit threshold effects. Specifically, values exceeding approximately 40 ms may substantially elevate the risk, whereas subthreshold values contribute minimally. This is a pattern that tree-based SHAP analysis would detect, but a linear odds ratio estimate would partially obscure (Li et al., 2011). Similarly, the protective effect of hamstring strength may be conditional on concurrent quadriceps strength (i.e., the H/Q interaction), which the random forest captures implicitly, whereas the main-effects logistic regression does not (Schmitt et al., 2012). Therefore, clinicians and researchers should treat these complementary analyses as jointly informative: logistic regression odds ratios provide interpretable, communicable effect size estimates predicated on linearity, whereas SHAP values offer a more comprehensive representation of feature contributions reflecting the full complexity of the data-generating process.

A third consideration is the moderate overall discriminative performance and consistently limited precision across all five algorithms. The peak AUC of 0.739 falls within the range conventionally regarded as ‘acceptable’ but below the 0.80 threshold often considered desirable for clinical prediction models (Hosmer et al., 2013). Precision values of 0.21–0.26 indicate that approximately three in four patients flagged as high-risk will not actually sustain a secondary injury. This limited precision has direct clinical implications: the model should not be used for making standalone clinical decisions or as a definitive individual-level warning system, but rather as a screening tool to identify patients who may benefit from closer monitoring, additional psychological assessment, or intensified rehabilitation. Several factors contribute to this performance ceiling. First, the events-per-variable ratio of approximately 2.1 (64 events across 30 predictors) falls well below the recommended minimum of 10–20 for stable model estimation, a fundamental statistical constraint that no algorithm can fully overcome (van Smeden et al., 2016). Second, secondary ACL injury is inherently multifactorial and partially stochastic, encompassing situational determinants such as contact mechanism, playing surface, and fatigue state, which are inaccessible at pre-participation clinical assessment (Hewett et al., 2016). Third, the 13.1% event rate mathematically constrains precision: even a highly discriminative model will generate substantial false positives when the prior event probability is low. Decision curve analysis demonstrated that the model’s utility is concentrated in the low-threshold screening range (0.05–0.15), supporting its use as a tool to identify patients warranting intensified monitoring rather than as a definitive diagnostic instrument. The high NPVs across all models (0.89–0.94) support this screening role, affording clinicians reasonable confidence that patients classified as low-risk have a genuinely low probability of secondary injury, whereas high-risk classifications require additional clinical judgment before being acted upon.

Fourth, return to sport was deliberately excluded from the predictor set. Return to sport showed a strong univariate association with secondary injury (*p* = 0.011) and would have improved discriminative performance if included; however, its inclusion would have introduced data leakage, as the predictor and outcome could not be temporally disentangled in a retrospective cohort design. Patients who resume athletic activity, particularly high-level pivoting sports, encounter biomechanical conditions conducive to secondary ACL injury, rendering the predictor-outcome boundary undefined (Grindem et al., 2018). In prospective clinical applications, secondary injury risk must be estimated prior to the return-to-sport decision, at which point return-to-sport status is not yet known. This distinction between associative and predictive modeling is critical for clinical applications. Future studies employing prospective longitudinal designs with clearly defined prediction horizons (e.g., predicting secondary injury within 24 months of ACLR using variables assessed at 6 months postoperatively) would resolve this temporal ambiguity and establish a more rigorous basis for evaluating the incremental predictive value of return-to-sport timing and intensity (Ardern et al., 2016).

The fifth set of considerations encompasses the methodological strengths and remaining limitations of this study. The nested cross-validation design with inner-loop hyperparameter tuning and strict per-fold pre-processing prevents information leakage between model selection and performance evaluation, yielding less optimistically biased estimates than simple k-fold cross-validation with external tuning (Varma and Simon, 2006). Platt scaling successfully reduced all Brier scores below the naïve baseline, resolving the calibration deficiency that would otherwise render the predicted probabilities clinically unreliable. The application of the Benjamini–Hochberg FDR procedure to the 30 univariate comparisons appropriately controls the multiple testing error rate (Benjamini and Hochberg, 1995). The addition of bootstrap confidence intervals for the AUC and domain ablation effects, together with SHAP stability analysis, provides quantitative assessments of estimation uncertainty that inform the confidence with which these findings should be interpreted. The primary limitations are the single-center retrospective design, which restricts generalizability to other surgical cohorts, graft types, and rehabilitation contexts, and the absence of an independent external validation cohort in the study. Therefore, external validation is the most critical next step according to the TRIPOD guidelines (Collins et al., 2015). The low events-per-variable ratio raises concerns about model stability, as evidenced by the wide confidence intervals for several odds ratios and the moderate (rather than high) bootstrap stability of the SHAP feature rankings. The single-timepoint assessment at 6 months postoperatively captures a snapshot of the patient’s status; psychological, strength, and gait characteristics may evolve between this assessment and the eventual injury event, potentially attenuating the predictive accuracy. Furthermore, the SF-36 Mental subscale was retained in the model despite its non-significant univariate difference (*p* = 0.226), a decision supported by the established principle that univariate non-significance does not preclude multivariate predictive contribution and that variable exclusion based on univariate screening introduces bias in multivariable prediction models (Sun et al., 1996).

In conclusion, this study demonstrated that a machine learning model integrating psychological, biomechanical, imaging, and strength assessments can identify patients at an elevated risk of secondary injury after ACLR with acceptable discrimination and calibration. The psychological domain provided the largest incremental predictive contribution among the five domains examined, with kinesiophobia and depressive symptoms consistently emerging as the top-ranked features in both linear and nonlinear models. These findings suggest that systematic psychological screening within postoperative rehabilitation may help identify patients at elevated risk, although the moderate overall performance and limited precision reflect the inherent difficulty of predicting rare, multifactorial events from cross-sectional data and highlight the necessity of external validation, prospective longitudinal designs, and larger multicenter cohorts with adequate event rates before clinical implementation. Future research should also investigate dynamic prediction models incorporating serial measurements of psychological and functional recovery trajectories, potentially leveraging wearable sensor data and ecological momentary assessments to capture the temporal evolution of risk throughout rehabilitation.

## Data Availability

The raw data supporting the conclusions of this article will be made available by the authors, without undue reservation.

## References

[ref1] Ardern C. L. Österberg A. Tagesson S. Gauffin H. Webster K. E. Kvist J. (2014). The impact of psychological readiness to return to sport and recreational activities after anterior cruciate ligament reconstruction. Br. J. Sports Med. 48, 1613–1619. doi: 10.1136/bjsports-2014-093842, 25293342

[ref2] Ardern C. L. Taylor N. F. Feller J. A. Whitehead T. S. Webster K. E. (2013). Psychological responses matter in returning to preinjury level of sport after anterior cruciate ligament reconstruction surgery. Am. J. Sports Med. 41, 1549–1558. doi: 10.1177/0363546513489284 23733635

[ref3] Ardern C. L. Glasgow P. Schneiders A. G. Witvrouw E. Clarsen B. Cools A. . (2016). Consensus statement on return to sport from the first world congress in sports physical therapy, Bern. Br. J. Sports Med. 50, 853–864. doi: 10.1136/bjsports-2016-096278 27226389

[ref4] Benjamini Y. Hochberg Y. (1995). Controlling the false discovery rate: a practical and powerful approach to multiple testing. J. R. Stat. Soc. Ser. B: Stat. Methodol. 57, 289–300. doi: 10.1111/j.2517-6161.1995.tb02031.x

[ref5] Biercevicz A. M. Akelman M. R. Fadale P. D. Hulstyn M. J. Shalvoy R. M. Badger G. J. . (2015). MRI volume and signal intensity of ACL graft predict clinical, functional, and patient-oriented outcome measures after ACL reconstruction. Am. J. Sports Med. 43, 693–699. doi: 10.1177/0363546514561435 25540298 PMC4344859

[ref6] Brewer B. W. Cornelius A. E. Sklar J. H. van Raalte J. L. Tennen H. Armeli S. . (2006). Pain and negative mood during rehabilitation after anterior cruciate ligament reconstruction: a daily process analysis. Scand. J. Med. Sci. Sports 17, 520–529. doi: 10.1111/j.1600-0838.2006.00601.x 17076828

[ref7] Collins G. S. Reitsma J. B. Altman D. G. Moons K. G. M. (2015). Transparent reporting of a multivariable prediction model for individual prognosis or diagnosis (TRIPOD): the TRIPOD statement. BMJ 350:g7594. doi: 10.1136/bmj.g7594 25569120

[ref8] Coronado R. A. Bird M. L. van Hoy E. E. Huston L. J. Spindler K. P. Archer K. R. (2018). Do psychosocial interventions improve rehabilitation outcomes after anterior cruciate ligament reconstruction? A systematic review. Clin. Rehabil. 32, 287–298. doi: 10.1177/0269215517728562 28836467

[ref9] Di Stasi S. L. Logerstedt D. Gardinier E. S. Snyder-Mackler, L. (2013). Gait patterns differ between ACL-reconstructed athletes who pass return-to-sport criteria and those who fail. Am. J. Sports Med. 41, 1310–1318. doi: 10.1177/0363546513482718, 23562809 PMC3732407

[ref10] Everhart J. S. Best T. M. Flanigan D. C. (2015). Psychological predictors of anterior cruciate ligament reconstruction outcomes: a systematic review. Knee Surg. Sports Traumatol. Arthrosc. 23, 752–762. doi: 10.1007/s00167-013-2699-1 24126701

[ref11] Grindem H. Arundale A. J. Ardern C. L. (2018). Alarming underutilisation of rehabilitation in athletes with anterior cruciate ligament reconstruction: four ways to change the game. Br. J. Sports Med. 52, 1162–1163. doi: 10.1136/bjsports-2017-098746, 29650520

[ref12] Grindem H. Snyder-Mackler L. Moksnes H. Engebretsen L. Risberg M. A. (2016). Simple decision rules can reduce reinjury risk by 84% after ACL reconstruction: the Delaware-Oslo ACL cohort study. Br. J. Sports Med. 50, 804–808. doi: 10.1136/bjsports-2016-096031, 27162233 PMC4912389

[ref13] Hartigan E. H. Lynch A. D. Logerstedt D. S. Chmielewski T. L. Snyder-Mackler L. (2013). Kinesiophobia after anterior cruciate ligament rupture and reconstruction: noncopers versus potential copers. J. Orthop. Sports Phys. Ther. 43, 821–832. doi: 10.2519/jospt.2013.4514, 24175594 PMC4915102

[ref14] Hewett T. E. Myer G. D. Ford K. R. Paterno M. V. Quatman C. E. (2016). Mechanisms, prediction, and prevention of ACL injuries: cut risk with three sharpened and validated tools. J. Orthop. Res. 34, 1843–1855. doi: 10.1002/jor.23414, 27612195 PMC5505503

[ref15] Hosmer D. W. Lemeshow S. Sturdivant R. X. (2013). Applied Logistic Regression. Wiley Series in Probability and Statistics. Hoboken: Wiley.

[ref16] Kunze K. N. Polce E. M. Rasio J. Nho S. J. (2021). Machine learning algorithms predict clinically significant improvements in satisfaction after hip arthroscopy. Arthroscopy 37, 1143–1151. doi: 10.1016/j.arthro.2020.11.027 33359160

[ref17] Kvist J. Ek A. Sporrstedt K. Good L. (2005). Fear of re-injury: a hindrance for returning to sports after anterior cruciate ligament reconstruction. Knee Surg. Sports Traumatol. Arthrosc. 13, 393–397. doi: 10.1007/s00167-004-0591-8 15703963

[ref18] Kyritsis P. Bahr R. Landreau P. Miladi R. Witvrouw E. (2016). Likelihood of ACL graft rupture: not meeting six clinical discharge criteria before return to sport is associated with a four times greater risk of rupture. Br. J. Sports Med. 50, 946–951. doi: 10.1136/bjsports-2015-095908 27215935

[ref19] Lentz T. A. Zeppieri G. Jr. George S. Z. Tillman S. M. Moser M. W. Farmer K. W. . (2015). Comparison of physical impairment, functional, and psychosocial measures based on fear of reinjury/lack of confidence and return-to-sport status after ACL reconstruction. Am. J. Sports Med. 43, 345–353. doi: 10.1177/0363546514559707, 25480833

[ref20] Li X. Kuo D. Theologis A. Carballido-Gamio J. Stehling C. Link T. M. . (2011). Cartilage in anterior cruciate ligament-reconstructed knees: MR imaging T1rho and T2--initial experience with 1-year follow-up. Radiology 258, 505–514. doi: 10.1148/radiol.10101006 21177392 PMC3029884

[ref21] Luc B. Gribble P. A. Pietrosimone B. G. (2014). Osteoarthritis prevalence following anterior cruciate ligament reconstruction: a systematic review and numbers-needed-to-treat analysis. J. Athl. Train. 49, 806–819. doi: 10.4085/1062-6050-49.3.35, 25232663 PMC4264654

[ref22] Lundberg S. M. Lee S.-I. (2017). A unified approach to interpreting model predictions. Adv. Neural Inf. Proces. Syst. 30, 4765–4774. doi: 10.5555/3295222.3295230

[ref23] Mall N. A. Chalmers P. N. Moric M. Tanaka M. J. Cole B. J. Bach B. R. Jr. . (2014). Incidence and trends of anterior cruciate ligament reconstruction in the United States. Am. J. Sports Med. 42, 2363–2370. doi: 10.1177/0363546514542796 25086064

[ref24] Martin R. K. Wastvedt S. Pareek A. Persson A. Visnes H. Fenstad A. M. . (2022). Machine learning algorithm to predict anterior cruciate ligament revision demonstrates external validity. Knee Surg. Sports Traumatol. Arthrosc. 30, 368–375. doi: 10.1007/s00167-021-06828-w, 34973096 PMC8866372

[ref25] Montalvo A. M. Schneider D. K. Yut L. Webster K. E. Beynnon B. Kocher M. S. . (2019). "what's my risk of sustaining an ACL injury while playing sports?" a systematic review with meta-analysis. Br. J. Sports Med. 53, 1003–1012. doi: 10.1136/bjsports-2016-096274, 29514822 PMC6561829

[ref26] Nordhausen K. (2009). The elements of statistical learning: data mining, inference, and prediction, second edition by Trevor Hastie, Robert Tibshirani, Jerome Friedman. Int. Stat. Rev. 77, 482–482. doi: 10.1111/j.1751-5823.2009.00095_18.x

[ref27] Paterno M. V. Rauh M. J. Schmitt L. C. Ford K. R. Hewett T. E. (2014). Incidence of second ACL injuries 2 years after primary ACL reconstruction and return to sport. Am. J. Sports Med. 42, 1567–1573. doi: 10.1177/0363546514530088, 24753238 PMC4205204

[ref28] Paterno M. V. Schmitt L. C. Ford K. R. Rauh M. J. Myer G. D. Huang B. . (2010). Biomechanical measures during landing and postural stability predict second anterior cruciate ligament injury after anterior cruciate ligament reconstruction and return to sport. Am. J. Sports Med. 38, 1968–1978. doi: 10.1177/0363546510376053, 20702858 PMC4920967

[ref29] Platt J. C. (2000). “Probabilities for SV machines,” in Advances in Large-Margin Classifiers, (Cambridge: The MIT Press), 61–74.

[ref30] Saito T. Rehmsmeier M. (2015). The precision-recall plot is more informative than the ROC plot when evaluating binary classifiers on imbalanced datasets. PLoS One 10:e0118432. doi: 10.1371/journal.pone.0118432, 25738806 PMC4349800

[ref31] Sanders T. L. Maradit Kremers H. Bryan A. J. Larson D. R. Dahm D. L. Levy B. A. . (2016). Incidence of anterior cruciate ligament tears and reconstruction: a 21-year population-based study. Am. J. Sports Med. 44, 1502–1507. doi: 10.1177/0363546516629944 26920430

[ref32] Schmitt L. C. Paterno M. V. Hewett T. E. (2012). The impact of quadriceps femoris strength asymmetry on functional performance at return to sport following anterior cruciate ligament reconstruction. J. Orthop. Sports Phys. Ther. 42, 750–759. doi: 10.2519/jospt.2012.4194, 22813542 PMC4157226

[ref33] Steyerberg E. W. Harrell F. E. Jr. (2016). Prediction models need appropriate internal, internal-external, and external validation. J. Clin. Epidemiol. 69, 245–247. doi: 10.1016/j.jclinepi.2015.04.005, 25981519 PMC5578404

[ref34] Sun G. W. Shook T. L. Kay G. L. (1996). Inappropriate use of bivariable analysis to screen risk factors for use in multivariable analysis. J. Clin. Epidemiol. 49, 907–916. doi: 10.1016/0895-4356(96)00025-X, 8699212

[ref35] van Smeden M. de Groot J. A. H. Moons K. G. M. Collins G. S. Altman D. G. Eijkemans M. J. C. . (2016). No rationale for 1 variable per 10 events criterion for binary logistic regression analysis. BMC Med. Res. Methodol. 16:163. doi: 10.1186/s12874-016-0267-3, 27881078 PMC5122171

[ref36] Varma S. Simon R. (2006). Bias in error estimation when using cross-validation for model selection. BMC Bioinform. 7:91. doi: 10.1186/1471-2105-7-91, 16504092 PMC1397873

[ref37] Vickers A. J. Elkin E. B. (2006). Decision curve analysis: a novel method for evaluating prediction models. Med. Decis. Mak. 26, 565–574. doi: 10.1177/0272989X06295361, 17099194 PMC2577036

[ref38] Webster K. E. Feller J. A. (2016). Exploring the high reinjury rate in younger patients undergoing anterior cruciate ligament reconstruction. Am. J. Sports Med. 44, 2827–2832. doi: 10.1177/0363546516651845, 27390346

[ref39] Webster K. E. Feller J. A. Lambros C. (2008). Development and preliminary validation of a scale to measure the psychological impact of returning to sport following anterior cruciate ligament reconstruction surgery. Phys. Ther. Sport 9, 9–15. doi: 10.1016/j.ptsp.2007.09.003, 19083699

[ref40] Wiggins A. J. Grandhi R. K. Schneider D. K. Stanfield D. Webster K. E. Myer G. D. (2016). Risk of secondary injury in younger athletes after anterior cruciate ligament reconstruction: a systematic review and Meta-analysis. Am. J. Sports Med. 44, 1861–1876. doi: 10.1177/0363546515621554, 26772611 PMC5501245

[ref41] Youden W. J. (1950). Index for rating diagnostic tests. Cancer 3, 32–35. doi: 10.1002/1097-0142(1950)3:1<32::AID-CNCR2820030106>3.0.CO;2-3 15405679

