# Supplementary material for: Psychological factors demonstrate the largest incremental predictive value in a multi-domain machine learning model for secondary injury risk after ACL reconstruction
Source: Front Psychol. 2026 May 15;17:1832229. doi: 10.3389/fpsyg.2026.1832229 (PMC13218976; doi:10.3389/fpsyg.2026.1832229)
Supplement: Supplementary file 1 [file Supplementary_File_1.docx]

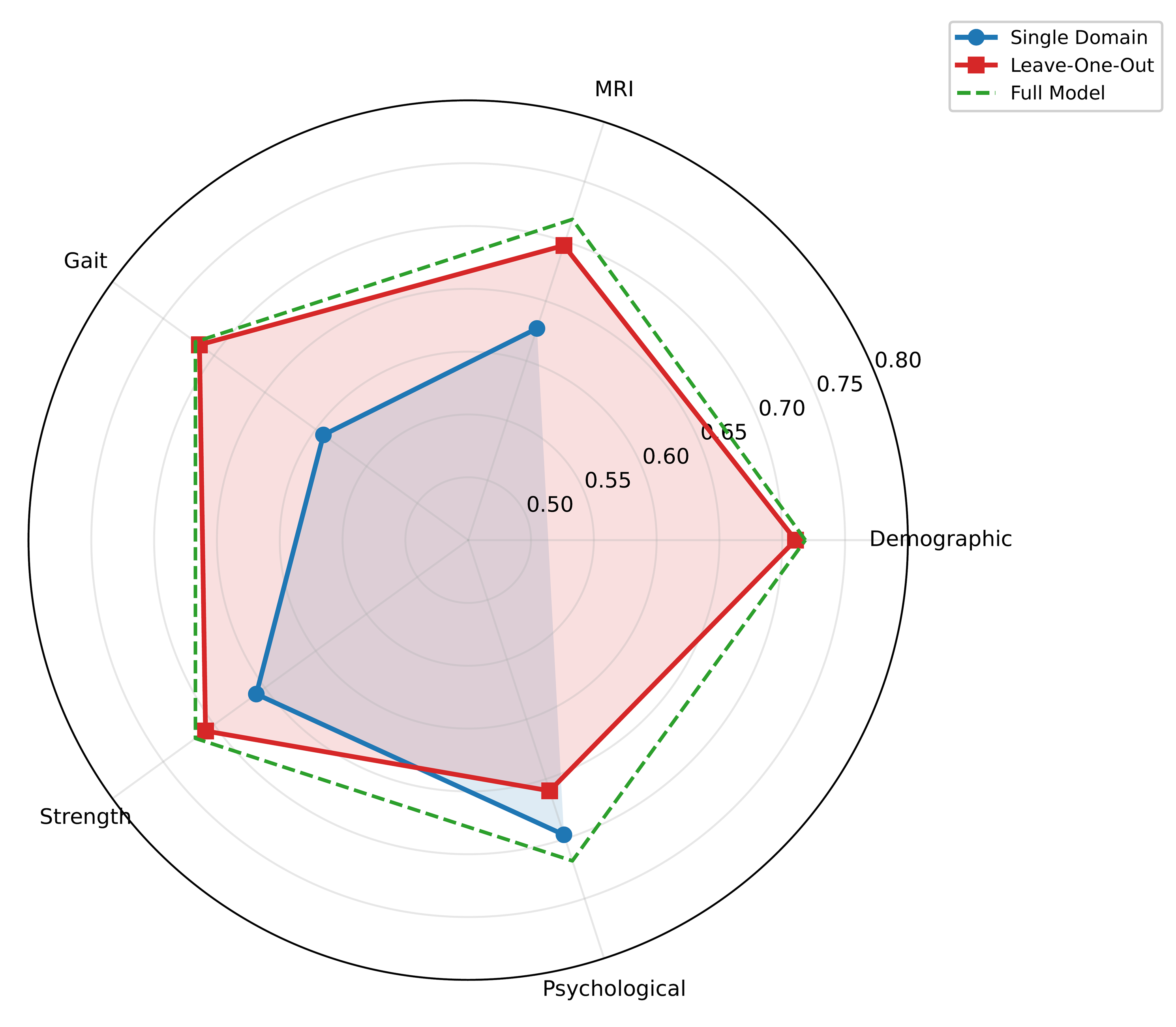


**Supplementary Figure S1.** Radar chart comparing five machine learning models across six performance metrics. Each axis represents a normalized performance dimension; values closer to the outer boundary indicate superior performance.
