# Supplementary material for: Psychological factors demonstrate the largest incremental predictive value in a multi-domain machine learning model for secondary injury risk after ACL reconstruction
Source: Front Psychol. 2026 May 15;17:1832229. doi: 10.3389/fpsyg.2026.1832229 (PMC13218976; doi:10.3389/fpsyg.2026.1832229)
Supplement: Supplementary file 2 [file Table_1.docx]

**Supplementary Table S1. Fold-level AUC values for all five models across five outer cross-validation folds.**

| **Fold** | **LR** | **RF** | **LightGBM** | **XGBoost** | **SVM** |
| --- | --- | --- | --- | --- | --- |
| 1 | 0.636 | 0.651 | 0.603 | 0.586 | 0.558 |
| 2 | 0.712 | 0.710 | 0.688 | 0.664 | 0.621 |
| 3 | 0.748 | 0.742 | 0.730 | 0.698 | 0.643 |
| 4 | 0.782 | 0.768 | 0.762 | 0.724 | 0.668 |
| 5 | 0.832 | 0.819 | 0.827 | 0.783 | 0.715 |
| Mean ± SD | 0.742 ± 0.072 | 0.738 ± 0.062 | 0.722 ± 0.082 | 0.691 ± 0.071 | 0.641 ± 0.057 |

*LR, logistic regression; RF, random forest; SVM, support vector machine. All values are from out-of-fold predictions in the outer loop of the nested cross-validation procedure.*

**Supplementary Table S2. Bootstrap stability of SHAP top-3 and top-5 feature rankings (500 resamples).**

| **Feature** | **RF: Top-3 (%)** | **RF: Top-5 (%)** | **LR: Top-3 (%)** | **LR: Top-5 (%)** |
| --- | --- | --- | --- | --- |
| TSK Score | 87.4 | 94.2 | 85.8 | 93.6 |
| PHQ-9 | 83.2 | 92.8 | 90.2 | 96.0 |
| Hamstring LSI | 71.6 | 85.4 | 52.4 | 74.8 |
| Loading Rate | 34.8 | 62.0 | 68.4 | 86.2 |
| MRI T2 Mapping | 48.6 | 72.6 | 28.2 | 51.4 |
| KOOS-QoL | 38.2 | 66.4 | 31.6 | 58.2 |
| H/Q Ratio | 22.4 | 48.8 | 46.8 | 72.4 |

*RF, random forest; LR, logistic regression; TSK, Tampa Scale for Kinesiophobia; PHQ-9, Patient Health Questionnaire-9; LSI, limb symmetry index; H/Q, hamstring-to-quadriceps; KOOS-QoL, Knee Injury and Osteoarthritis Outcome Score quality-of-life subscale. Percentages indicate the proportion of 500 bootstrap resamples in which each feature appeared among the top-3 or top-5 ranked features by mean |SHAP| value.*

**Supplementary Table S3. Collinearity filtering: excluded variables, collinear pairs, and rationale for variable retention (|r| > 0.70 threshold, within-domain Spearman correlations).**

| **Excluded Variable** | **Retained Variable** | **Domain** | **Spearman \|r\|** | **Rationale for Retention** |
| --- | --- | --- | --- | --- |
| Graft Signal Intensity | MRI T2 Mapping | MRI | 0.76 | T2 mapping provides quantitative compositional tissue characterization |
| Bone Edema Volume | Tunnel Widening | MRI | 0.74 | Tunnel widening is a more standardized and reproducible radiographic measurement |
| Cartilage Thickness | MRI T2 Mapping | MRI | 0.72 | T2 mapping captures compositional changes beyond morphological thickness |
| Gait Asymmetry Index | Step Length Difference | Gait | 0.82 | Step length difference is more directly clinically interpretable |
| Peak Knee Flexion | Vertical Loading Rate | Gait | 0.73 | Loading rate reflects dynamic weight acceptance more relevant to injury mechanism |
| Peak Valgus Moment | Step Length Difference | Gait | 0.71 | Step length difference retained as primary inter-limb asymmetry metric |
| Quadriceps LSI | Hamstring LSI | Strength | 0.78 | Hamstring LSI showed stronger univariate association with secondary injury |
| Hamstring Peak Torque | Quadriceps Peak Torque | Strength | 0.75 | Quadriceps deficit more clinically emphasized in post-ACLR rehabilitation |
| ACL-RSI | TSK Score | Psychological | 0.78 | TSK is more widely established in re-injury risk literature |
| Sex | Tegner Pre-injury† | Demographic | 0.74 | Tegner captures pre-injury activity level more directly than sex |
| Tegner Pre-injury† | Age | Demographic | 0.73 | Age is a fundamental confounder with broader clinical relevance |
| Meniscus Injury | Cartilage Injury | Demographic | 0.72 | Cartilage injury has greater structural implications for secondary injury risk |
| BMI | Smoking† | Demographic | 0.72 | Smoking has direct physiological effects on graft healing and tissue quality |
| Graft Type | Time to Surgery† | Demographic | 0.74 | Time to surgery captures injury-to-reconstruction interval relevant to tissue recovery |
| Time to Surgery† | Age | Demographic | 0.71 | Age is a more fundamental predictor with broader applicability |

*† Variables that appear both as “Excluded” and as “Retained” in different rows reflect sequential filtering: the variable was initially retained from one pair but was subsequently excluded due to a higher correlation with a different variable. LSI, limb symmetry index; ACL-RSI, ACL-Return to Sport after Injury scale; TSK, Tampa Scale for Kinesiophobia; BMI, body mass index.*
